# Shining fresh light on the evolution of photosynthesis

**DOI:** 10.7554/eLife.01403

**Published:** 2013-09-28

**Authors:** Areejit Samal, Olivier C Martin

**Affiliations:** 1 **Areejit Samal** is at the International Center for Theoretical Physics, Trieste, Italy; 2 **Olivier C Martin** is at the UMR de Génétique Végétale, Institut National de la Recherche Agronomique, Gif-sur-Yvette, Franceolivier.martin@moulon.inra.fr

**Keywords:** Convergent evolution, C4 photosynthesis, Bayesian model, Other

## Abstract

There are two types of photosynthesis, C3 and C4, and computational techniques have been used to explore how C4 plants evolved from their C3 ancestors.

**Related research article** Williams BP, Johnston IG, Covshoff S, Hibberd JM. 2013. Phenotypic landscape inference reveals multiple evolutionary paths to C_4_ photosynthesis. *eLife* **2**:e00961. doi: 10.7554/eLife.00961

**Image** Different evolutionary paths showing traits being added to an initial phenotype (bottom left) in different orders

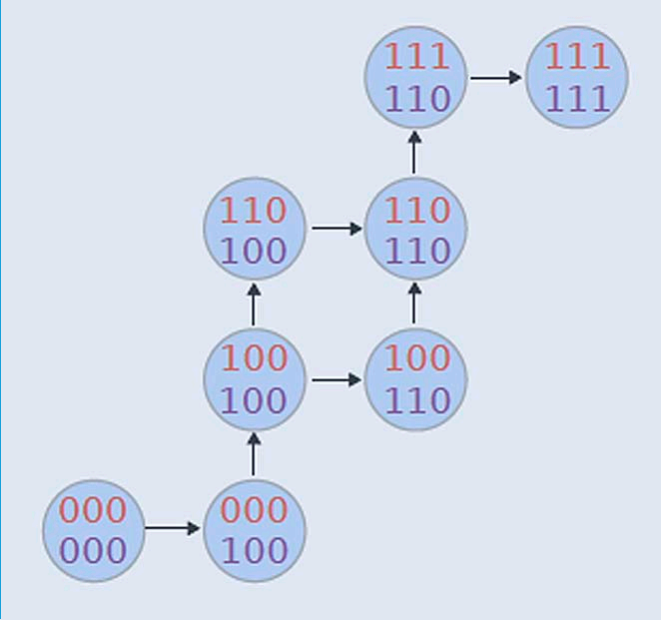

Convergent evolution is the independent appearance of similar traits or biological innovations in different evolutionary lineages. Classic cases include the acquisition of wings in insects, birds and bats, or the emergence of camera-eyes in cephalopods and vertebrates. One of the most dramatic examples of convergent evolution is seen in photosynthesis—the process that plants use to capture energy from the sun and convert carbon dioxide and water into carbohydrates and oxygen. The most common form of photosynthesis is called C3 photosynthesis, but some plants have developed a more efficient form of this process called C4 photosynthesis.

The convergent evolution of C4 photosynthesis is remarkable for two reasons. First, it involved changes at many different length scales: for example, it involved changes in carbon metabolism, which occurs on the molecular scale, and changes in the structure of cells and tissues. Second, it has appeared in a huge number of lineages, including more than 60 for land plants alone. Now, in *eLife*, researchers from Cambridge University and Imperial College—including Ben Williams and Iain Johnston as joint first authors—report that they have used computational modelling to gain fresh insights into the evolution of C4 photosynthesis in plants (Williams et al., 2013).

At the heart of the power of plants to convert carbon dioxide into carbohydrate is an enzyme called RuBisCO, which is the most abundant protein on earth (Figure 1A). RuBisCO incorporates carbon dioxide into the Calvin-Benson cycle; this process, which is also known as carbon fixation, leads to the synthesis of biomass and thus to the growth of the plant. However, RuBisCO also reacts with oxygen in a process called photorespiration, and the competition between photosynthesis and photorespiration in C3 plants has a negative impact on their growth.

**Figure 1.**
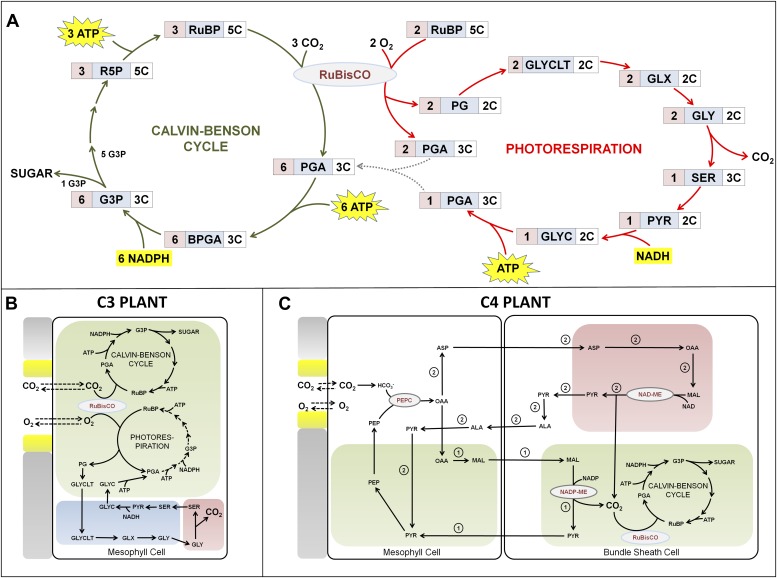
Photosynthesis in C3 and C4 plants. (**A**) There is competition between the Calvin-Benson cycle (photosynthesis) and photorespiration in C3 plants because the enzyme RuBisCO (ribulose-1,5-bisphosphate carboxylase oxygenase) is involved in both processes. When carbon dioxide is plentiful, the Calvin-Benson cycle converts carbon dioxide and water into carbohydrate (sugar) and oxygen: the energy needed to drive this process comes from the sun via ATP and NADPH molecules. However, when levels of carbon dioxide are low, photorespiration consumes energy and carbon dioxide without producing any carbohydrates. Acronyms are spelt out at the end of the caption: the number of carbon atoms in a molecule of the metabolite is given on the right, and the number of molecules involved in the reaction is on the left. (**B**) In C3 plants the Calvin-Benson cycle and photorespiration both occur in mesophyll cells, which are next to the pores (shown in yellow) that allow the plant to exchange gases with the atmosphere (and also allow water vapour to escape from the plant). Different organelles within the cells are illustrated in different colours: chloroplasts in green; peroxisome in blue; mitochondria in pink. (**C**) In C4 plants mesophyll cells absorb atmospheric gases and harness energy from the sun using chlorophyll, while the fixation of carbon dioxide by RuBisCO generally takes place in bundle sheath cells, which have no direct interactions with the atmosphere. Two variations of the C4 cycle are shown: reactions unique to the enzyme NADP-ME are labelled 1, and those unique to enzyme NAD-ME are labelled 2. Acronyms: ALA, ASP, GLY and SER are all amino acids; PEPC (phosphoenolpyruvate carboxylase) is an enzyme; the other metabolites are BPGA (1,3-bisphosphoglycerate), G3P (glyceraldehyde 3-phosphate), GLYC (glycerate), GLYCLT (glycolate), GLX (glyoxylate), MAL (malate), OAA (oxaloacetate), PEP (phosphoenolpyruvate), PG (phosphoglycolate), PGA (3-phosphoglycerate), PYR (pyruvate), R5P (ribulose 5-phosphate) and RuBP (ribulose-1,5-bisphosphate).

In C4 plants, however, photorespiration is minimized by using compartmentation to ensure that RuBisCO can no longer react with oxygen. The majority of C4 plants implement this via cell specialization: mesophyll cells absorb atmospheric gases through pores in the leaves of the plant, while bundle sheath cells use RuBisCO to fix the carbon dioxide (Figure 1B,C). Carbon is shuttled back and forth between the cells by molecules that contain four carbon atoms, hence the name C4 photosynthesis.

This physical separation means that the RuBisCO enzyme is exposed to higher concentrations of carbon dioxide, which reduces the need to exchange carbon dioxide and oxygen with the atmosphere. Thus the plant can close the pores that allow these gases—and water vapour—to enter and leave the plant. This, in turn, keeps the loss of water to a minimum and helps the plant to resist drought: it is widely thought that the ability to survive droughts was important for the evolutionary emergence of C4 plants (Tipple and Pagnani, 2007; Kadereit et al., 2012). Much of the interest in C4 photosynthesis is motivated by the goal of transferring the higher rates of photosynthesis and growth found in C4 plants, along with their reduced need for water, to C3 plants such as rice.

To understand how C4 plants could have evolved from C3 plants, the Cambridge-Imperial team—which also includes Sarah Covshoff and Julian Hibberd—explored how C4 plants acquired a number of the traits that distinguish them from C3 plants. The 16 traits included in the study ranged from differences in the ways that cells accumulated various enzymes to differences in their size. The study exploited data from 72 plant species, including a number with phenotypes that are intermediate between C3 and C4.

The team used computational and statistical techniques to infer the probabilities of evolutionary trajectories that go from C3 to C4 phenotypes. In their framework, the probability of a trajectory can be calculated in terms of the probability of going from an arbitrary intermediate phenotype to a phenotype with one extra trait (see image on page 1). Since these ‘transition probabilities’ are unknown, Williams, Johnston et al. took a Bayesian approach and sampled these probabilities. In practice, this involved applying a technique known as Markov Chain Monte Carlo to adjust the set of transition probabilities to make them compatible with the experimental data—in this case the experimental data were the intermediate C3–C4 phenotypes.

Once formulated, this mathematical framework allowed questions about patterns in evolutionary trajectories to be answered objectively, with no human biases to skew the conclusions. Williams, Johnston et al. wanted to know if the evolutionary trajectories were strongly constrained (Lobkovsky et al., 2011; Heckmann et al., 2013). To answer this question they needed to know if the C3–C4 intermediate phenotypes had emerged from more than one evolutionary path. The answer to this question was yes. Next they asked in what order do C4 traits tend to be acquired. Interestingly, they found clear patterns: any given trait tends to be acquired at a characteristic time but, nonetheless, the order in which traits are acquired is not strict, which leads to a large amount of flexibility in the evolutionary trajectories. Williams, Johnston et al. also found that there were different classes of trajectories associated with the two major groups of flowering plants (monocots vs. eudicots).

What forces cause certain evolutionary trajectories to be more likely than others in the emergence of C4 plants? Selective pressures that favoured drought resistance and carbon capture in spite of high levels of oxygen were certainly present, but Williams, Johnston et al. give evidence that other forces may also have had a role. Furthermore, it is quite plausible that simple traits are acquired faster than more complex traits. Indeed, the acquisition of new traits typically involves modifying regulatory circuits and recruiting existing molecules to perform new functions (Barve and Wagner, 2013), which will require fewer changes for some traits than others.

It will take time to perform the sort of in-depth molecular investigation that is needed to clarify the details of how the different traits were acquired. In addition to testing some of the conclusions reported by Williams, Johnston et al., such experiments would also be of help to researchers trying to transfer C4 traits to C3 crops, be it via genetic engineering or breeding programmes using well chosen C3–C4 intermediates.
